# The first compound heterozygous mutations in SLC12A3 and PDX1 genes: a unique presentation of Gitelman syndrome with distinct insulin resistance and familial diabetes insights

**DOI:** 10.3389/fendo.2023.1327729

**Published:** 2024-01-25

**Authors:** Yaqi Yin, Liqin Li, Songyan Yu, Yu Xin, Lili Zhu, Xiao Hu, Kang Chen, Weijun Gu, Yiming Mu, Li Zang, Zhaohui Lyu

**Affiliations:** ^1^ Department of Endocrinology, The First Medical Center of Chinese People’s Liberation Army General Hospital, Beijing, China; ^2^ Department of Endocrinology, Baoding No. 1 Central Hospital, Baoding, China; ^3^ Department of Endocrinology, Beijing Tiantan Hospital, Capital Medical University, Beijing, China; ^4^ School of Medicine, Nankai University, Tianjin, China; ^5^ Department of Endocrinology and Cardiology, TaiYuan No.8 People Hospital, Taiyuan, China; ^6^ Department of Internal Medicine, The 63790th Hospital of Chinese People’s Liberation Army, Xichang, China

**Keywords:** Gitelman syndrome, SLC12A3, PDX1, MODY4, insulin resistance

## Abstract

**Background:**

Gitelman Syndrome (GS) patients frequently exhibit disrupted glucose metabolism, attributed to hypokalemia, hypomagnesemia and heightened aldosterone. This study delved into the genetic underpinnings linked to insulin resistance and diabetes in a GS patient, contextualized within his family history.

**Methods:**

The hydrochlorothiazide and furosemide loading test were performed to ascertain the presence of GS. Oral glucose tolerance test (OGTT) evaluated glucose metabolism and insulin sensitivity. Whole-exome sequencing, validated by Sanger sequencing, was employed to confirm gene mutations, which were then tracked among the patient’s relatives.

**Results:**

Symptoms and laboratory examination confirmed the clinical diagnosis of GS. Comprehensive whole-exome sequencing, augmented by Sanger sequencing validation, revealed a compound heterozygous mutation within the SLC12A3 gene (c.1108G>C in exon 9, c.676G>A in exon 5 and c.2398G>A in exon 20) in the patient. The OGTT affirmed diabetes and heightened insulin resistance, distinct from previous patients with GS we evaluated. Further genetic analysis identified a missense heterozygous mutation (c.97C>G in exon 1) within the PDX1 gene, inherited from the patient’s diabetic mother without GS. Furthermore, the patient’s brother, with impaired glucose tolerance but regular potassium levels, also bore this mutation, hinting at additional impacts of the PDX1 gene mutation on glucose metabolism regulation beyond the known impacts of GS.

**Conclusion:**

This study unveils unprecedented compound heterozygous mutations in the SLC12A3 and PDX1 genes in a GS patient. These findings illuminate the potential complex genetic factors influencing glucose metabolism disruptions in GS.

**Take-home message:**

This research uncovers a novel combination of SLC12A3 and PDX1 gene mutations in a Gitelman Syndrome patient, revealing intricate genetic factors that potentially disrupt glucose metabolism and shedding light on familial diabetes links.

## Introduction

Gitelman syndrome (GS) is a distinctive autosomal-recessive disorder marked by hypokalemic metabolic alkalosis, hypomagnesemia, hypocalciuria, and a secondary surge in the renin-angiotensin-aldosterone system (RAAS). Often, it presents with regular or diminished blood pressure. At its genetic foundation lies the Solute Carrier Family 12 Member 3 (SLC12A3) gene (MIM No.600968), responsible for encoding the thiazide-sensitive sodium-chloride co-transporter (NCC) (1, 2). Positioned on the apical membranes of the distal convoluted tubule (DCT) cells, this transporter is pivotal in regulating ion transport. Recent research has unveiled a multitude of novel mutations in the SLC12A3 gene (3–5). And the Human Gene Mutation Database (HGMD) has documented close to 500 mutations of the SLC12A3 gene (6), spanning types like missense, nonsense, frame-shift, and splice-site, distributed throughout the protein.

Chronic hypomagnesemia and hypokalemia, hallmark traits of GS patients, can disrupt glucose metabolism by affecting insulin secretion and sensitivity (2). Notably, GS patients exhibit a higher prevalence of diabetes mellitus compared to the general population. Some studies have even indicated that the degree of insulin resistance in GS patients may parallel that observed in individuals with type 2 diabetes (T2D) (7). The study conducted by Tao Yuan and colleagues (7) demonstrated that GS patients have higher serum glucose and insulin levels post-glucose intake compared to healthy controls, yet these levels are lower than those in T2D patients. Additionally, the insulin secretion-sensitivity index for GS patients lies between that of healthy individuals and T2D patients. In another study focusing on a smaller group of Chinese GS patients (8), researchers found elevated glucose and insulin levels post-glucose loading, along with a delayed peak in insulin secretion compared to healthy controls. This study further highlighted the significantly higher insulin resistance in GS patients, as determined by the homeostasis model assessment, and their relatively lower insulin sensitivity indices. Despite these connections, comprehensive investigations into glucose metabolism within GS patients remain limited, and the precise interplay between hypokalemia, hypomagnesemia, and aberrant glucose metabolism necessitates further exploration.

Maturity-onset diabetes of the young (MODY) constitutes a diverse group of disorders marked by diabetes mellitus, typically inherited in an autosomal dominant manner, with diagnosis frequently occurring before the age of 25, often during childhood or adolescence, and characterized by a primary defect in β-cell function (9). A rare variant of MODY, known as MODY4, is linked to mutations in PDX1, a pivotal transcription factor in pancreatic and β-cell development and function (10, 11). Remarkably, our study uncovered a GS patient who not only presented with diabetes but also displayed heightened insulin resistance compared to other GS patients. This individual’s family history of diabetes added an intriguing layer to the puzzle. Driven by these compelling observations, we initiated an extensive investigation culminating in the identification of a compound heterozygous mutation within the SLC12A3 gene (c.1108G>C in exon9, c.676G>A in exon5), alongside a mutation within the Pancreatic and Duodenal Homeobox 1 (PDX1) gene, for this specific patient.

## Materials and methods

### Ethical approval

Approval for this research was obtained from our institution’s ethics committee (S2022-014-01). The study design strictly met the ethical standards set forth in the Helsinki Declaration. Given the study’s minimal risk, a waiver for obtaining informed consent from participants was granted.

### Study population

For comparative analysis, we evaluated GS patients admitted to the Endocrinology Department of the Chinese PLA General Hospital, Beijing, China, from January 2012 to December 2021. In accordance with the expert consensus for the diagnosis and treatment of patients with GS (12), an initial cohort of 54 GS patients was identified. Following the exclusion of patients lacking either glycated hemoglobin A1C (HbA1c) or oral glucose tolerance test (OGTT) data, a total of 46 patients were ultimately included.

### Laboratory measurements

Serum and 24-hour urinary concentrations of electrolytes, creatinine, and glucose were assessed through an automated analyzer (Cobas 8000 Modular Analyzer series; Roche Diagnostics, Basel, Switzerland). HbA1c, insulin, and C-peptide levels were quantified using a high-performance liquid chromatography on the VARIANT II Hemoglobin Testing System (Tosoh Corporation, Tokyo, Japan). Blood gas analysis was performed using the RAPIDPoint 500 blood gas analyzer (Siemens, Germany). Insulin resistance was assessed through the Homeostasis Model Assessment of Insulin Resistance Index (HOMA-IR), calculated as fasting insulin (μU/ml) × fasting glucose (mmol/L) ÷ 22.5. Measurement of plasma aldosterone and direct plasma renin levels was conducted through chemiluminescence immunoassay, with analysis facilitated using the LIAISONX Analyzer. All laboratory procedures were conducted in the Biochemistry Department and the Endocrine Laboratory of the Chinese PLA General Hospital.

### Clinical examinations and definitions

#### Oral glucose tolerance test

After a 12hour overnight fast, patients underwent OGTT by consuming 75 g of glucose in 250-300 mL of water within 5 minutes. Plasma glucose, C-peptide, and insulin levels were measured at 0, 60, and 120 minutes post-consumption.

#### Upright renin-angiotensin-aldosterone system test

After lying supine for at least 4 hours, patients stood upright for 4 hours. Blood samples were taken at the 2-hour and 4-hour marks during the standing phase to evaluate plasma renin and aldosterone levels using radioimmunoassay.

#### Hydrochlorothiazide test and the furosemide loading test

The hydrochlorothiazide (HCT) test was executed following a standardized protocol as previously outlined (13). Prior to the test, patients were instructed to discontinue spironolactone for a minimum of 7 days and halt potassium and magnesium supplementation one day ahead of time. After an overnight fast, patients assumed a supine position and consumed 10 ml of water per kilogram of body weight 15 minutes before the test. Subsequently, they ingested 150 ml of water per hour until the test concluded. Following two initial 30 minute baseline clearance periods, either HCT (50 mg orally) or furosemide (20 mg intravenously) was administered, followed by six additional 30 minute clearance periods. Blood samples were obtained at 60 and 240 minutes, and urine samples were collected every 30 minutes for electrolyte and creatinine analysis. The quantification of chloride excretion was achieved through fractional excretion (FE, utilizing creatinine as a GFR marker) utilizing the formula: FECl = (UCl/SCl) × (SCr/UCr) × 100%, where SCr and UCr denote serum and urinary creatinine, respectively. For GS diagnosis, the blunt HCT test was defined as a net increase in chloride fractional excretion (ΔFECl) below 2.86% (12).

### Genomic DNA extraction and sequence analysis

#### Target region capture and sequencing

A peripheral blood sample of 5 ml was collected from the patient for genomic DNA (gDNA) extraction. The extraction procedure followed the standard protocol provided by the manufacturer (MagPure Buffy Coat DNA Midi KF Kit). Subsequently, BGI’s enzyme kit (Segmentase, BGI) was employed to fragment the gDNA into segments ranging from 100 to 500 base pairs. From these fragments, segments measuring 280-320 base pairs were selectively collected using magnetic beads. To facilitate adapter attachment, “A” bases were introduced at the 3’ overhangs following repair. This ensured compatibility for pairing with a specific adapter featuring a “T” base. Subsequently, a single individual DNA library was created through LM-PCR (ligation-mediated polymerase chain reaction) and purification processes. The library underwent a 16-24 hours enrichment phase (at 47°C) through array hybridization (Roche NimbleGen, Madison, USA), followed by elution and post-capture amplification. The resulting products were analyzed using the Agilent 2100 Bioanalyzer and BMG to gauge the extent of enrichment. Once qualified, the library products were pooled and quantified based on their individual library quantities. The library products’ single strands were prepared for circularization to create DNA nanoballs (DNBs). Subsequently, the samples were subjected to sequencing with PE100 + 100 on the MGISEQ-2000 platform.

#### Bioinformatics analyses and data processing

In order to identify potential variants within the patient’s genetic makeup, we undertook a series of bioinformatics analyses and data processing subsequent to receiving the initial sequencing data. Employing established filtering criteria (14), we curated a set of “clean reads” from the sequencing data, each consisting of 90 base pairs. These curated “clean reads” were then aligned to the human genome reference (hg19) using the BWA (Burrows Wheeler Aligner) Multi-Vision software package (15). Upon alignment, the resultant output files underwent comprehensive analysis. This analysis included assessing sequencing coverage and depth within the targeted region, as well as identifying single-nucleotide variants (SNVs) and insertions/deletions (INDELs). To identify SNVs and indels, we employed the GATK software (16). To enhance accuracy, all identified SNVs and indels were subjected to filtration and cross-referenced with multiple databases, such as NCBI dbSNP, HapMap, the 1000 Human Genome Dataset, and the Database of 100 Chinese Healthy Adults. For assessing the potential impact of missense variants, we turned to dbNSFP (17), a resource housing seven widely recognized in silico prediction tools including Polyphen2, MutationTaster, and SIFT. To classify pathogenic variants, we adhered to the protocol outlined by the American College of Medical Genetics and Genomics (ACMG) (18). Furthermore, we consulted the HGMD to screen for mutations previously reported in published studies. For the annotation of mutation sites identified in our study, we utilized the online protein topology visualization tool Protter (http://wlab.ethz.ch/protter/start/). Additionally, the SWISS-MODEL software (https://swissmodel.expasy.org/) was employed to predict the three-dimensional structures of the proteins in question.

#### Sanger verification

Conventional Sanger sequencing methods were employed to validate all identified mutations and potential pathogenic variants. In cases where DNA samples from family members were accessible, segregation analysis was carried out to further confirm the findings.

## Results

### Clinical and biochemical characteristics

A 38-year-old Chinese male presented with a two-year history of unexplained fatigue, nocturia, and thirst. A recent hospitalization for pneumonia in a local facility identified hypokalemia (2.2 mmol/L). The patient was treated with 5.0-6.0 g/day of oral potassium chloride sustained-release tablets, anti-infection, and phlegm-resolving therapy, which ameliorated symptoms and stabilized blood potassium levels between 2.69-3.35 mmol/L. Due to the persistent low potassium levels, he was referred to our endocrine department.

The patient’s blood pressure was measured at 112/81 mmHg. His measurements for waist circumference, hip circumference, and BMI were 92 cm, 100 cm, and 25.1 kg/m^2^, respectively. Laboratory analysis (**Table 1**) revealed hypokalemia, hypochloremia, and blood magnesium at the lower limit of the normal range. The patient’s arterial blood pH was alkaline. Urine tests revealed alkalinity with elevated renal potassium and chloride excretion, and a decreased urine calcium-to-creatinine ratio of 0.18. The 24 hour urine protein was within the normal range. The patient’s electrocardiogram showed sinus rhythm, left axis deviation, and T-wave changes in leads I, aVL, II, and V4-V6. Comprehensive assessments involving cardiopulmonary, abdominal, and urinary ultrasounds, as well as neurological examinations and adrenal computed tomography, revealed no abnormalities.

**Table 1:** Main laboratory tests of the patient pre- and post-treatment.

Variable	Pre-treatment Test value	Post-treatment Test value	Normal range
Blood tests			
Serum Creatinine (umol/L)	83.1	82.7	63.65-104.31
Fasting blood glucose (mmol/L)	7.03	5.9	3.4-6.1
HbA1c (%)	6.7	6.1	4-6
Serum Sodium (mmol/L)	140.2	136	130-150
Serum Potassium (mmol/L)	2.5	3.55	3.5-5.5
Serum Choride (mmol/L)	91.7	95	94-110
Serum Calcium (mmol/L)	2.3	2.46	2.09-2.54
Serum Phosphorus (mmol/L)	0.89	1.28	0.89-1.6
Serum Magnesium (mmol/L)	0.67	0.87	0.6-1.4
Serum Albumin (g/dL)	43.8	42.6	35-50
Blood PH	7.47	7.44	7.35-7.45
PCO_2_ (mmHg)	42.7	39.9	35-45
HCO_3_- (mmol/L)	27.5	25.6	20-26
BE (mmol/L)	3.2	2.9	-3-3
Urine tests			
Urinary pH	7.0	–	5.0-8.0
24h urinary volume (ml)	3100	–	
Urine potassium (mmol/24h)	128.65	–	25-100
Urine sodium (mmol/24h)	223.2	–	130-260
Urine magnesium (mmol/24h)	6.2	–	2.1-8.2
Urine chloride (mmol/24h)	258.85	–	170-250
Urine creatinine (mmol/24h)	13.33	–	7.0-17.6
Urine calcium/creatinine (mmol/mmol)	0.18	–	>0.2
Urine protein (g/L)	0.07	–	<0.150

HbA1c, glycosylated hemoglobin A1C; PH, potential of hydrogen; PCO_2_, partial pressure of carbon dioxide; BE, base excess.

With evident low blood potassium levels combined with an increase in urinary potassium excretion, the possibility of renal potassium loss in the patient was evident. While the patient maintained normal blood pressure and exhibited metabolic alkalosis, it became essential to contemplate the potential diagnoses of GS, Bartter syndrome, or diuretic usage. Notably, the patient had no history of diuretic utilization, and it’s important to highlight that Bartter syndrome primarily manifests in infants and young children, whereas GS is more prevalent among adults. Given this context, the elevated likelihood of GS prompted the necessity for further differentiation through hydrochlorothiazide and furosemide testing. Experimental outcomes indicated a net increase of ΔFECl by 0.652% following hydrochlorothiazide administration, and a surge by 14.659% post intravenous furosemide injection. These findings signified the patient’s heightened sensitivity to furosemide and strongly supported the diagnosis of GS (**Table 2**). The outcomes of the upright RAAS test revealed notable activation of the patient’s RAAS, accompanied by a significant increase in renin levels during the supine position. Subsequent transition to an upright position further amplified the renin levels, consistent with the characteristic manifestation of GS (**Table 3**).

**Table 2:** Results of HCT test and the furosemide loading test.

Item	HCT test			Furosemide test		
FEX%	FEX _Pre-test_	FE _Maximum_	△_FEX_	FEX _Pre-test_	FE _Maximum_	△_FEX_
FEK(%)	19.535	19.919	0.384	18.590	68.5	49.91
FENa(%)	0.754	1.552	0.798	0.500	10.658	10.158
FECL(%)	1.311	1.963	0.652	0.862	15.521	14.659
FEMg(%)	3.242	3.447	0.205	3.252	17.270	14.018
FEP(%)	7.257	10.656	3.399	9.773	11.507	1.734
FECa(%)	0.356	0.406	0.050	0.293	5.547	5.254

HCT, Hydrochlorothiazide; FEK, fractional excretion of potassium; FENa, fractional excretion of sodium; FEP, fractional excretion of phosphorus; FECa, fractional excretion of calcium; FEMg, fractional excretion of magnesium; FECl, fractional excretion of chloride; ΔFEX, the net increase in X (K/Na/P/Ca/Cl) fractional excretion.

**Table 3:** Results of upright RAAS test.

Time	Direct renin (uIU/mL)	Aldosterone (ng/dl)	Aldosterone to Renin Ratio
0	159.7	10	0.1
2h	267.6	11.4	<0.1
4h	276.0	9.2	<0.1

RAAS, renin-angiotensin-aldosterone system.

**Figure 1:**
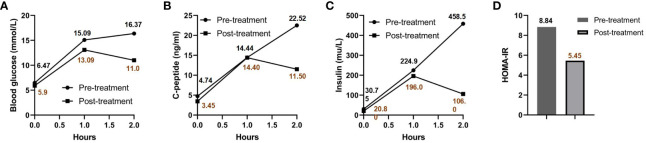
OGTT and HOMA-IR results pre- and post-treatment. Blood glucose **(A)**, C-peptide **(B)**, and insulin levels **(C)** are charted at various intervals post-sugar intake, both prior to and following treatment. The patient’s HOMA-IR before and after treatment is presented in **(D)**.

**Figure 2:**
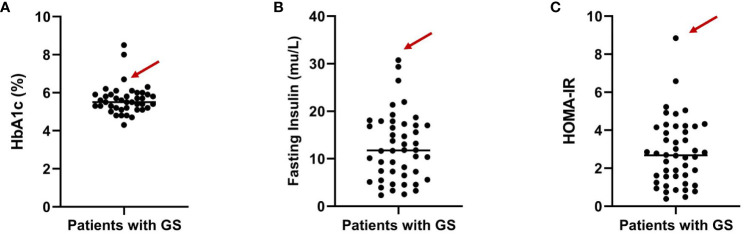
Distribution of HbA1c levels **(A)**, fasting insulin **(B)** and HOMA-IR **(C)** among GS patients from our department. The arrows indicate the specific value for the highlighted patient.

Impaired glucose tolerance is a common characteristic among patients with GS. In this particular case, the patient presented with a fasting blood glucose level exceeding 7 mmol/L and an HbA1c > 6.5% upon admission. In order to gain further insights into the patient’s blood glucose profile, an OGTT was administered (**Figures 1A–C**). The results unveiled that the patient’s blood glucose level measured 2 hours after glucose ingestion exceeded 11.1 mmol/L, satisfying the diagnostic criteria for diabetes. Furthermore, the patient exhibited a fasting insulin level of >20 mU/L, with insulin levels soaring to 458.5 mU/L 2 hours after glucose intake, and a HOMA-IR of 8.84 (**Figure 1D**), signifying a notable degree of insulin resistance. For comparative analysis, we collected data from 46 GS patients in our department, including 30 males (65.2%) and 16 females (34.8%), with an average age of 34.7 ± 13.6 (Q1-Q3: 24.2-43.0) years old, BMI of 25.1 ± 3.6 kg/m^2^ (Q1-Q3: 22.3-27.5), blood potassium levels of 2.8 ± 0.4 mmol/L (Q1-Q3: 2.6-3.1), blood magnesium levels of 0.6 ± 0.2 mmol/L (Q1-Q3: 0.5-0.8), HbA1c levels of 5.6 ± 0.8% (Q1-Q3: 5.2-5.9) (**Figure 2A**), fasting insulin of 12.4 ± 7.0 mu/L (Q1-Q3: 6.7-17.0) (**Figure 2B**) and HOMA-IR of 2.8 ± 1.7 (Q1-Q3: 1.6-4.1) (**Figure 2C**). Remarkably, this patient exhibited the highest HOMA-IR value and the most severe insulin resistance among this population, despite a relatively average weight and non-critically low magnesium and potassium levels. The patient’s parents and brother did not exhibit hypokalemia, yet the patient’s mother had a 15-year history of diabetes, initiating insulin therapy during the fifth year of her diabetes diagnosis. Additionally, the patient’s brother exhibited impaired glucose tolerance. Regarding the exocrine pancreas, the patient’s pancreatic enzyme levels were within the normal range (serum lipase 26.3 U/L (13-60) and serum amylase 53.8 U/L (0-150)). Additionally, no signs of pancreatic dysfunction were evident in the patient, his mother, or younger brother. In light of these familial associations, the patient underwent genetic testing to ascertain the presence of gene abnormalities that could potentially relate to both the hypokalemia and diabetes phenotype.

### Mutations of the SLC12A3 and PDX1 genes

**Figure 3:**
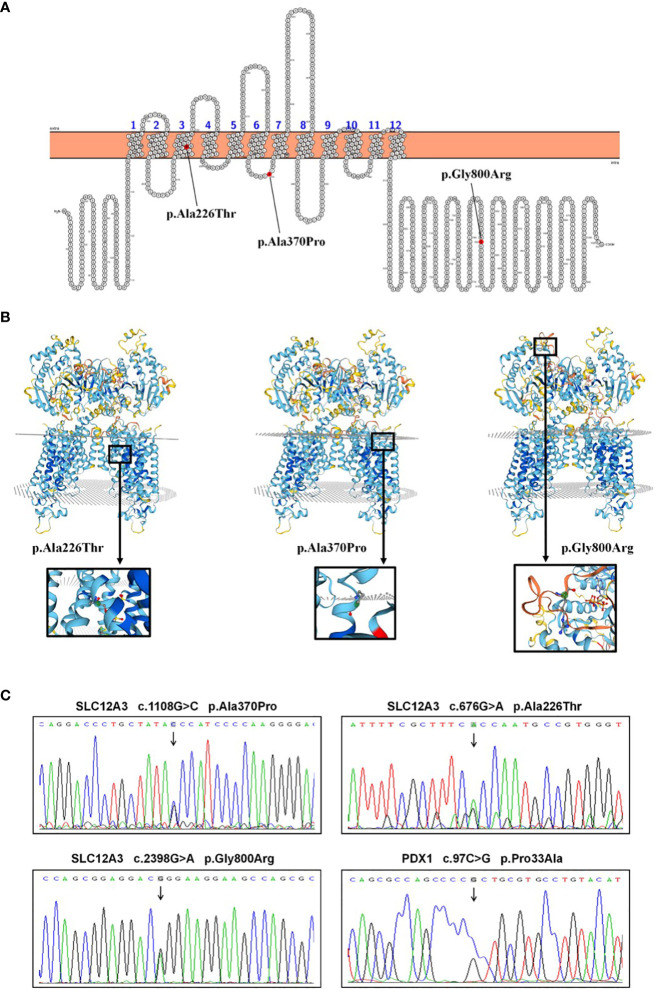
SLC12A3 and PDX1 mutations: localization, 3D structure, and DNA sequencing analysis. **(A)** Localization of SLC12A3 mutations on the NCC protein structure. The diagram shows the intracellular amino- and carboxy-terminal domains, along with transmembrane domains 1 to 12. The SLC12A3 mutation sites are indicated, with mutations from left to right being p.Ala226Thr, p.Ala370Pro, and p.Gly800Arg. **(B)** The predictive three-dimensional structures of the NCCT with missense mutations. From left to right, the diagrams depict the three-dimensional structures of the NCC with p.Ala226Thr, p.Ala370Pro, and p.Gly800Arg mutations. The mutation sites are highlighted within a black box, magnified and presented in a spherical bar chart format. **(C)** DNA sequence analysis of SLC12A3 and PDX1 missense mutations identified in the patient. Arrows indicate heterozygous nucleotide substitutions. Sequencing at the 1108th base of the 9th exon of SLC12A3 revealed a G-to-C substitution, resulting in an amino acid substitution from Ala to Pro at position 370. Sequencing at the 676th base of the 5th exon of SLC12A3 showed a G-to-A substitution, leading to an amino acid substitution from Ala to Thr at position 226. Sequencing at the 2398th base of the 20th exon of SLC12A3 demonstrated a G-to-A substitution, causing an amino acid substitution from Gly to Arg at position 800. Sequencing at the 97th base of the 1st exon of PDX1 indicated a C-to-G substitution, resulting in an amino acid substitution from Pro to Ala at position 33.

**Figure 4:**
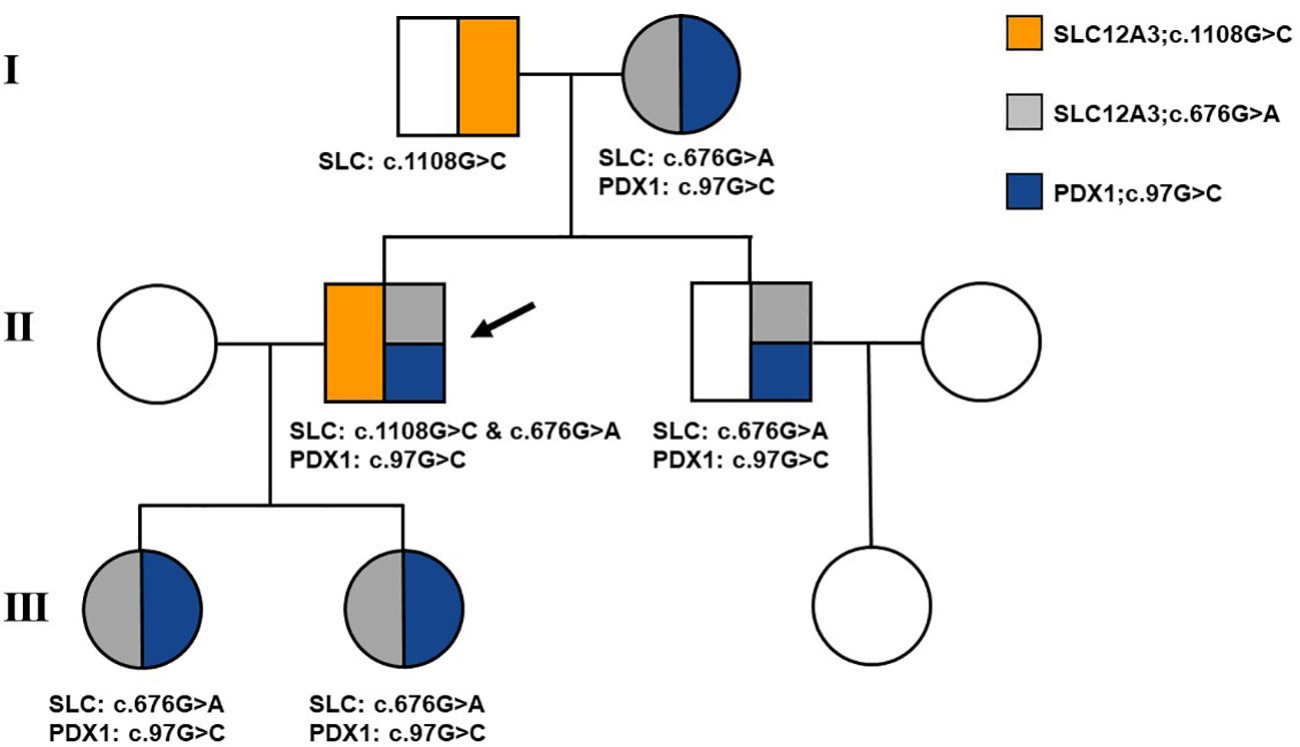
Family pedigree of the patient. Affected individuals are represented by solid symbols. The arrow indicates the patient.

Following the acquisition of written informed consent from the patient and his family, genomic DNA sequencing was undertaken. The sequencing identified the patient as a compound heterozygote with three distinct mutations in the SLC12A3 gene: c.1108G>C, c.676G>A, and c.2398G>A. Sequencing at the 1108th base of the 9th exon of SLC12A3 revealed a G-to-C substitution, resulting in an amino acid substitution from Ala to Pro at position 370. Sequencing at the 676th base of the 5th exon of SLC12A3 showed a G-to-A substitution, leading to an amino acid substitution from Ala to Thr at position 226. Sequencing at the 2398th base of the 20th exon of SLC12A3 demonstrated a G-to-A substitution, causing an amino acid substitution from Gly to Arg at position 800. The detailed mutation site distribution map of the NCC is presented in **Figure 3A**, and the predictive three-dimensional structural diagrams for the missense mutations are displayed in **Figure 3B**. The p.Ala370Pro mutation is located in the transmembrane region of NCC, while the other two mutation sites are in the intracellular segment of the protein. **Figure 3C** presents the DNA sequence analysis of SLC12A3, showing the specific nucleotide substitutions and resulting amino acid changes. Using computational prediction tools, we assessed the potential implications of these mutations, with findings consolidated in **Table 4**. Our analysis pinpointed the first two mutations, which translated into the missense variants p.Ala370Pro and p.Ala226Thr at the protein level, as likely contributors to the pathogenic features of GS. It’s significant to note that previous studies have associated these mutations with the disease’s pathogenesis (19–21). The third mutation, however, was deduced to be benign. Sanger sequencing-based family verification revealed the inheritance pattern of these mutations: the SLC12A3 c.676G>A mutation was maternally inherited, while the remaining two were paternally derived (depicted in **Figure 4**). Adding another layer of complexity, the patient also harbored a mutation in the PDX1 gene: c.97C>G in exon 1 (**Figure 3C**). This mutation, deemed pathogenic via software evaluation, aligns with existing literature affirming its pathogenicity (22). This PDX1 mutation was traced back to the patient’s mother and was also identified in his younger brother. Furthermore, the patient transmitted both the SLC12A3 c.676G>A and PDX1 c.97C>G mutations to his two daughters (as shown in **Figure 4**), neither of whom currently manifest hypokalemia or abnormal glucose tolerance. Collectively, these genetic discoveries provide a compelling narrative that connects the dots between the patient’s diabetes, pronounced insulin resistance, and the family’s diabetes and impaired glucose tolerance history.

**Table 4:** Comprehensive details of patient’s unique mutations and corresponding predictive outcomes from various tools.

Gene	SLC12A3	SLC12A3	SLC12A3	PDX1
RNA	NM_001126108.1	NM_001126108.1	NM_001126108.1	NM_000209.3
Exon	EX5	EX9	EX20	EX1
DNA mutation	c.676G>A	c.1108G>C	c.2398G>A	c.97C>G
AA mutation	p.Ala226Thr	p.Ala370Pro	p.Gly800Arg	p.Pro33Ala
Population frequency (ExAC)	0.000008	Unknown	0.000091	0.000156
Polyphen	0.998 **Probably damaging**	0.997 **Probably damaging**	0.001 Benign	0.996 **Probably damaging**
MutationTaster Pred	**disease causing**	**disease causing**	Polymorphism automatic/harmless	**disease causing**
SIFT	Unknown	Unknown	Unknown	**Deleterious**

### Treatment and follow-up

The patient was prescribed a regimen of oral supplements, consisting of potassium magnesium aspartate (316 mg/280 mg, 3 times daily), potassium chloride (2000 mg, 3 times daily), and spironolactone (20 mg, 1 time daily). Alongside medication, a diet rich in potassium was advised. To regulate blood glucose levels, Metformin was administered at a dose of 500 mg, three times daily. After a rigorous treatment period spanning 2.5 years, a detailed follow-up assessment was performed. This assessment covered essential metrics (**Table 1**) such as blood potassium (3.55 mmol/L), blood magnesium (0.87 mmol/L), blood sodium (136 mmol/L), blood chloride (95 mmol/L), and HbA1c (6.1%), all of which collectively suggested effective disease management. Interestingly, a significant improvement was observed when Metformin was discontinued for a week before re-administering the OGTT (as detailed in **Figures 1A–C**). The subsequent assessment unveiled a marked reduction in insulin resistance, highlighted by a HOMA-IR score of 5.45 (**Figure 1D**). This result intriguingly verifies that apart from the hypothesized effects of the PDX1 gene mutation, other contributing elements, such as decreased potassium and magnesium levels characteristic of GS, play a pivotal role in the multifaceted development of insulin resistance. It’s worth highlighting that, despite this improvement, the patient’s insulin resistance remained considerably higher compared to most individuals with GS.

## Discussion

The molecular basis of GS was elucidated in the late 19th century when Simon et al. (23) discovered a link between GS and the renal NCC, a membrane protein with 1030 amino acids, encompassing 12 transmembrane domains and intracellular N and C-terminals. This led to the identification of the human SLC12A3 gene encoding the NCC. Located on chromosome 16q, this gene spans roughly 55 kb and contains 26 unique exons. Most GS patients, being an autosomal recessive disorder, show homozygous or compound heterozygous mutations in SLC12A3. Biallelic inactivating mutations in this gene are diagnostic hallmarks of GS. However, certain GS patients with just one detected mutated allele via direct sequencing were later found to possess large genomic rearrangements on the other allele (24). To detect such cases, multiplex ligation-dependent probe amplification (MLPA) is advised. Moreover, mutations in the SLC12A3 intron or other genes, like the CLCNKB gene linked with Bartter syndrome, might be alternative molecular defects. A case exhibiting mutations in both SLC12A3 and CLCNKB genes suggests a digenetic inheritance pattern attributed to a dual genetic hit mechanism (25). In this patient of our study, genetic analysis unveiled compound heterozygous mutations of the SLC12A3 gene, specifically c.1108G>C in exon 9, c.676G>A in exon 5, and c.2398G>A in exon 20. The c.676G>A mutation was inherited from the patient’s mother, while c.1108G>C and c.2398G>A mutations were from his father. Notably, the c.1108G>C mutation in exon 9 and c.676G>A mutation in exon 5 have been predicted to be pathogenic, whereas c.2398G>A in exon 20 is considered non-pathogenic. The patient’s mother, father, brother, and two daughters did not exhibit any clinical symptoms of GS due to their heterozygous status. Clinical reports have demonstrated that function-loss mutations in the SLC12A3 gene, particularly including p.D486N, p.T60M, c.506-1G>A, p.S710X, c.2548 + 253C>T, and p.C430G, contribute to the pathogenic effects observed in GS. Notably, among Chinese patients with GS, the p.D486N and p.T60M variant emerge as the most prevalent mutation (19, 26). However, the mutation site identified in this patient is not a commonly observed hotspot mutation among the Chinese or Asian population.

GS manifests through chronic hypomagnesemia and hypokalemia, which in turn disrupt glucose metabolism due to impaired insulin secretion and sensitivity (2). Hypokalemia may disrupt the closure of ATP-sensitive potassium channels and L-type calcium channels on pancreatic β cell surfaces, precipitating insulin secretion impairments (27). And a low-potassium diet (40 mmol/d) has been shown to induce insulin secretion defects and reduced insulin sensitivity in healthy subjects (28). Ren et al. noted heightened oral glucose responses in 16 GS patients compared to 12 healthy subjects, with notable elevation of plasma glucose and insulin levels after glucose administration in the GS group, peaking at 2 hours post-administration (8). Blanchard et al. observed significantly elevated HOMA-IR indices in GS patients compared to healthy subjects (27). The pivotal role of magnesium as an enzymatic cofactor in energy production underlies its ability to influence insulin secretion and insulin receptor tyrosine kinase activity (29). Furthermore, the modulation of insulin secretion or sensitivity can influence hypomagnesemia, as insulin plays a crucial role in regulating the renal Mg^2+^ channel TRPM6 (30, 31). Notably, the subset of GS patients marked by severe hypomagnesemia tend to exhibit a higher insulin resistance index and a propensity for elevated BMI, regardless of severe hypokalemia (27). Additionally, GS patients may experience increased insulin resistance due to elevated aldosterone levels (32), which dose-dependently affects the insulin signaling pathway. In our study, we observed substantial improvement in insulin resistance, as evaluated by OGTT, and a reduction in HOMA-IR from 8.84 to 5.45 following symptomatic potassium and magnesium supplementation of the patient. These findings align with mechanisms identified in previous studies. Nevertheless, our patient presents unique attributes compared to other GS cases encountered in our department. The overweight patient diagnosed with diabetes exhibits pronounced insulin resistance even when blood potassium and magnesium levels are not severely depleted. Additionally, the patient has a familial history of diabetes. Thus, we explored whether factors beyond GS contribute to the aggravated insulin profile. As a result, we chose to comprehensively sequence all exons rather than exclusively screen for the SLC12A3 gene associated with GS, uncovering the presence of a PDX1 gene mutation.

Homozygous and compound heterozygote mutations in the PDX1 gene have been associated with permanent neonatal diabetes and exocrine pancreatic insufficiency due to pancreatic agenesis (33–35). On the other hand, heterozygous carriers of PDX1 mutations are linked to MODY4, characterized by modest glucose intolerance due to compromised insulin secretion. While individuals with type 2 diabetes commonly exhibit obesity, MODY patients typically maintain a lean profile (9). However, sporadic cases of obesity and hyperinsulinemia have been reported in certain MODY families. For instance, within an HNF-4α/MODY1 pedigree, obesity was observed in two diabetic members, one being a 7-year-old with fasting plasma glucose of 203 mg/dL and fasting insulin of 27 µU/mL (36). Similarly, a 13.5-year-old obese diabetic boy from Israel (BMI 29.8 kg/m^2^, blood glucose 417 mg/dL, HbA1c 11.8%) with an HNF-1α/MODY3 gene mutation exhibited elevated fasting C-peptide level of 1,489 pmol/L (37). A Czech study associated diabetes with a NeuroD1/MODY6 gene mutation in two families, all members of which were obese, with proband displaying elevated fasting C-peptide level (38). The original description of the NeuroD1/MODY6 form of diabetes (39) also reported obesity and “relatively high insulin levels” in diabetic members of a family. Within a five-generation Michigan-Kentucky pedigree, originating from a proband with pancreatic agenesis and homozygous for the PDX1 mutation Pro63fsx60, both the proband’s parents exhibited fasting hyperglycemia (155 and 147 mg/dL, respectively), elevated post-glucose plasma glucose levels (265 and 160 mg/dL, respectively), significantly increased fasting insulin (98 and 39 µU/mL, respectively) and C-peptide (8.0 and 5.0 ng/mL, respectively) levels, along with elevated postprandial levels (insulin 284 and 100 µU/mL, C-peptide 21.4 and 13.7 ng/mL, respectively) (40). Heterozygous carriers of MODY4 mutations within this pedigree were diagnosed at ages ranging from 2.5 years to their forties. Consequently, obesity, obesity-induced insulin resistance, and hyperinsulinemia may be a common occurrence in various MODY subtypes, suggesting at least a partial compensatory response that is not significantly compromised at the time of study. This underlines phenotypic similarities between obese MODY and type 2 diabetic patients, discernible mainly through genetic assessment. The degree of obesity within a specific population may vary across races or ethnicities (41). For instance, a prior study reported that approximately 10%-15% of Japanese children with T2D are non-obese (42). Our patient, who endured both diabetes and GS, was overweight according to Chinese population standards (19), and exhibits abdominal obesity. Preliminary OGTT outcomes pointed towards severe insulin resistance. Following extended treatment with potassium and magnesium supplementation, a subsequent OGTT showed marked improvement in insulin resistance, though it still persists to some extent with a HOMA-IR of 5.45. This suggested the patient may be in the early stages of diabetes, with mild functional abnormalities due to mutations at the corresponding site of PDX-1. The pancreatic islets’ β-cell function remained partially compensatory, although the emergence of GS initially intensified insulin resistance. Given the patient’s mother was diagnosed with diabetes in her mid-forties and transitioned to insulin-based therapy 5 years post-diagnosis, ongoing monitoring of the patient, their sibling, and children for changes in blood glucose, insulin resistance, and pancreatic function is crucial.

## Conclusion

In conclusion, our research elucidated a distinctive case of a GS patient exhibiting profound insulin resistance and bearing a family history of diabetes. Through meticulous exon sequencing, we pinpointed a mutation in the PDX1 gene, augmenting the previously detected anomaly in the SLC12A3 gene. This key finding emphasizes the consequential role of PDX1 gene mutations in the onset of diabetes and associated insulin resistance. Consequently, we’ve unveiled a novel pathway leading to altered glucose tolerance in GS patients, deepening our understanding of the complex interaction between genetic markers and metabolic disruptions. This novel insight offers a promising foundation for future studies delving into the multifaceted interconnections of diabetes, insulin resistance, and GS.

## Data availability statement

The original contributions presented in the study are included in the article, further inquiries can be directed to the corresponding authors.

## Ethics statement

This study involving human participants was reviewed and approved by the Ethics Committee of the Chinese PLA General Hospital. It adhered to the relevant local legislation and institutional guidelines. Written informed consent was obtained from all participants, and in the case of minors, from their legal guardians, for their participation and for the publication of potentially identifiable information contained in this article.

## Author contributions

YY: Writing – original draft, Writing – review & editing. LL: Writing – original draft. SY: Writing – review & editing. YX: Writing – original draft. LLZ: Writing – original draft. XH: Writing – review & editing. KC: Writing – review & editing. WG: Writing – review & editing. YM: Writing – review & editing. LZ: Writing – review & editing. ZL: Writing – review & editing.
